# mSphere of Influence: Expanding the CRISPR Sphere with Single-Locus Proteomics

**DOI:** 10.1128/mSphere.00001-20

**Published:** 2020-01-22

**Authors:** Lucy Glover

**Affiliations:** a Department of Parasites and Insect Vectors, Institut Pasteur, Paris, France

**Keywords:** *Trypanosoma brucei*, antigenic variation, VSG, DNA repair and recombination, CRISPR-Cas, DNA repair, recombination

## Abstract

Lucy Glover’s research focuses on the role of DNA repair and recombination in antigenic variation in the parasite Trypanosoma brucei, the causative agent of both human and animal African trypanosomiasis. In this mSphere of Influence article, she reflects on how **“**A CRISPR-based approach for proteomic analysis of a single genomic locus” by Z. J. Waldrip, S. D. Byrum, A. J. Storey, J. Gao, et al. (Epigenetics 9:1207–1211, 2014, https://doi.org/10.4161/epi.29919) made an impact on her research by taking the precision of CRISPR-Cas9 and repurposing it to look at single-locus proteomics. By using this technology in trypanosomes, Dr. Glover and her colleagues could study the dynamic accumulation of repair proteins after specific damage and gain insight into how the location of a double-strand break (DSB) dictates repair pathway choice and how this may influence immune evasion in these parasites.

## Abstract (precis)

Lucy Glover’s research focuses on the role of DNA repair and recombination in antigenic variation in the parasite Trypanosoma brucei, the causative agent of both human and animal African trypanosomiasis. In this mSphere of Influence article, she reflects on how **“**A CRISPR-based approach for proteomic analysis of a single genomic locus” by Z. J. Waldrip, S. D. Byrum, A. J. Storey, J.

## COMMENTARY

It appears that if you can imagine it, CRISPR can do it. Clustered regularly interspaced short palindromic repeats (CRISPR)−CRISPR-associated protein 9 (Cas9)-mediated genome modification is a type II CRISPR system from Streptococcus pyogenes that is the basis for prokaryote adaptive immunity (1) and that has quickly become a precision genome editing tool targeting either DNA or RNA (2) with a myriad of potential uses. Cas9 in its active form is an endonuclease that cleaves DNA and is able to bind to its target via a guide RNA (gRNA). However, by introducing mutations into the two Cas9 nuclease domains, researchers were able to retain the binding capacity of Cas9 but without DNA cleavage (3). This resulted in a CRISPR-deactivated Cas9 (dCas9) system that can be used to alter the transcription status of specific loci (4) or live-cell imaging of specific loci (5) and to interrogate long-range chromatin interactions (6). In the article “A CRISPR-based approach for proteomic analysis of a single genomic locus” by Waldrip et al. (7), the authors used CRISPR-dCas9 to develop CRISPR-chromatin affinity purification with mass spectrometry (ChAP-MS) technology to determine the changes in the local epiproteome, defined as posttranslational modifications (PTMs) of histones and other proteins, of a promoter during activation of transcription. Using CRISPR-ChAP-MS, the authors observed enrichment of chromatin from the *GAL1* gene promoter under activating conditions and identified a specific cohort of proteins associated with transcription. Although in our lab we do not study promoter activation, we are interested in the accumulation of proteins involved in DNA damage and repair after a double-strand break (DSB) and how this influences the ability of Trypanosoma brucei to exchange genes, specifically the variant surface glycoprotein (VSG) genes, required for immune evasion. Of the kinetoplastid parasites, T. brucei is the most well studied and genetically tractable (8), making new technologies potentially applicable to studies in this parasite. Could we use CRISPR-ChAP-MS to target specific loci after a DSB to interrogate the dynamics of repair and recombination?

Chromatin immunoprecipitation (ChIP)-MS has long been established as a method to define chromatin composition at specific regions in the cell (9), but it relies on a known protein for immunoprecipitation. CRISPR-ChAP-MS extends ChAP-MS and TAL (TALEN [transcription activator-like effector nuclease])-ChAP-MS approaches, both of which use quantitative high-resolution mass spectrometry to unambiguously identify specific PMTs and proteins localized to the region of interest. This technology uses dCas9 tagged with protein A (PrA) and targeted with a gRNA specific to the *GAL1* gene promoter region. The ability to activate the *GAL1* gene promoter in a synchronous manner by supplying Saccharomyces cerevisiae with galactose instead of glucose allowed the authors to target transcriptionally active chromatin for analysis. Formaldehyde-fixed, sheared chromatin was affinity purified using the PrA tag and subjected to label-free mass spectrometry. The CRISPR-ChAP-MS method was shown to have two benefits: (i) the improved unbiased target chromatin isolation at 1-kb resolution and (ii) the ease with which gRNAs can be designed for multiplexing, making this approach cost-effective. One disadvantage is that both CRISPR-ChAP-MS and TAL-ChAP-MS, which require binding to the DNA, are unlikely to be able to target repressed regions of chromatin.

Homologous recombination (HR) is a dynamic process that is essential for maintaining genomic integrity and for immune evasion in trypanosomes. Antigenic variation in trypanosomes is mainly via *VSG* gene conversion (GC) events, which are dependent on HR. We know that DSBs can trigger a *VSG* gene switch and that several DNA damage proteins are important for this process, but what constitutes this dynamic repair factory that forms and leads to switching is unknown (10). There are several features that could make implementing the method as described in the Waldrip et al. paper (7) in trypanosomes, to look at accumulation of DNA repair proteins, particularly attractive. First, similar to activation of the *GAL1* promoter, we are able to induce a single DSB in 95% of the population using the I-SceI meganuclease (10), which allows us to spatially and temporally control the accumulation of DNA damage repair proteins. Second, a single *VSG* gene is expressed from a specialized locus, called an expression site, at the telomeric end of a chromosome, making the chromatin we are interested in open. Third, DNA repair and recombination are dynamic processes meaning that we could assay the composition of the repair factory over multiple time points using the same gRNAs. The one challenge we would face is finding multiple potential gRNA sequences for tilling without relying wholly on a drug resistance cassette inserted into the target site, as in the trypanosome genome, there are approximately 20 expression sites which exhibit a high degree of homology. However, this is a problem that could be overcome. Understanding how trypanosomes switch the expressed *VSG* gene is not fully understood even though it is fundamental to their immune evasion strategy. A method that could allow us to sample, in an unbiased manner, the factors that leads to *VSG* gene switching is an exciting one.
